# Single-cell sequencing of brain tissues reveal the central nervous system’s susceptibility to SARS-CoV-2 and the drug

**DOI:** 10.3389/fphar.2022.971017

**Published:** 2022-09-13

**Authors:** Zhichao Lu, Ziheng Wang, Zhuhuan Song, Chen Chen, He Ma, Peipei Gong, Yunzhao Xu

**Affiliations:** ^1^ Department of Clinical Biobank, Affiliated Hospital of Nantong University, Nantong, China; ^2^ Department of Neurosurgery, Affiliated Hospital of Nantong University, Nantong, China; ^3^ Department of Neurosurgery, Aviation General Hospital, Beijing, China; ^4^ The Comprehensive Cancer Centre of Nanjing Drum Tower Hospital, The Affiliated Hospital of Nanjing University Medical School and Clinical Cancer Institute of Nanjing University, Nanjing, China; ^5^ Medical School of Soochow University, Suzhou, China

**Keywords:** SARS-CoV-2, single-cell, epigallocatechin, CNS, ACE2, catechins

## Abstract

**Background:** The severe acute respiratory syndrome coronavirus 2 (SARS-CoV-2) caused the current COVID-19 pandemic, resulting in a public health crisis that required immediate action. The SARS-CoV-2 virus enters human cells via three receptors, namely cathepsin, angiotensin-converting enzyme 2 (ACE2) and SARS-CoV receptors. Cathepsin destroys the spike protein (S protein), thereby allowing the entry of viral nucleic acid into human host cells.

**Methods:** Utilizing single-cell transcriptome analysis of brain tissues, the vulnerability of the central nervous system to infection with SARS-CoV-2 in humans was investigated.

**Results:** ACE2 is mainly expressed in endothelial cells, with the highest levels found in ageing endothelial cells. Drug prediction suggests that (-)-catechin reduces the effects of COVID-19 on the nervous system. Immunohistochemistry analysis showed that ACE2 was mainly expressed in cerebral vessels. Immunofluroscenceresults showed the co-expression of CD31 and ACE2 in human tissues. Western blot further showed that ACE2 expression was higher in old rats than in young rats.

**Conclusion:** This study provides insight into the mechanism of SARS-CoV-2 brain invasion. Accordingly, patients with neurological symptoms who are infected with SARS-CoV-2 should be given individualised care.

## Introduction

During the early days of December 2019, a novel transmittable infection of severe acute respiratory syndrome coronavirus 2 (SARS-CoV-2) spread rapidly across China (Ladner et al., 2020). The World Health Organization (WHO) classified SARS-CoV-2 as a worldwide viral pandemic on 11 March 2020 (Lambertini et al., 2021; Omolaoye et al., 2021). Millions of individuals globally have been impacted by the SARS-CoV-2 virus. SARS-CoV-2 infection is a heterogeneous illness (Bhattacharyya and Thelma, 2020; Mavian et al., 2020), with extensive clinical characteristics, such as asymptomatic infection, septic shock, acute respiratory distress syndrome (ARDS), mild upper respiratory tract infection, multi-organ failure and mortality (Boras et al., 2021).

Respiratory viral infections, like other forms of viral infections, invade the central nervous system (CNS) via the hematoma or various neural retrograde routes. In terms of the CNS, it is infiltrated by a viral agent via the circulatory system. Moreover, certain viruses can invade neurons in the peripheral nervous system and then utilizes the axonal transport mechanism to obtain entry into the CNS (Schwerk et al., 2015; Dahm et al., 2016a). In the hematoma pathway, a virus can successfully invade the endothelium of the blood-brain barrier (BBB) or the epithelium of the blood-cerebrospinal fluid barrier (BCSFB) in the choroid plexus (CP), which is located in the ventricular system of the brain, or leukocytes, which can serve as a vector for dispersion towards the CNS (Argyris et al., 2007; Atluri et al., 2015). Furthermore, numerous SARS-COV-2-infected individuals experienced signs of neurological symptoms such as vomiting, nausea and headache. A clear association between these symptoms and unfavourable outcomes has been widely reported. Additionally, Moriguchi et al. presented the very first incidence of encephalitis/meningitis correlated with SARS-CoV-2 infection in the cerebrospinal fluid (CSF) that did not result in a positive nasal polyp test (Moriguchi et al., 2020). Furthermore, it is unclear if SARS-CoV-2 can infiltrate the CSF or the CNS of asymptomatic individuals. Nonetheless, the vulnerability of human CNS cells to SARS-CoV-2 and its specific pathogenic processes remain largely unknown.

Catechins, a category of phenolics that are predominantly found in foodstuffs, including cocoa, tea leaves, vegetables, fruits and wine, have been well recognized for their intriguing health-promoting functions, such as antioxidative, antibiotic, neuroprotective, anti-inflammatory, and anticarcinogenic functions. As a result, green tea is one of the richest and most available catechin sources, containing (−)-epigallocatechin-3-gallate (EGCG), (−)-epigallocatechin (EGC), (−)-epicatechin-3-gallate (ECG) and (−)-epicatechin (EC) predominantly (Wang et al., 2021). These compounds have the capacity to destroy and also inhibit the spread of pathogenic pathogens. Numerous investigations have shown that EGCG suppresses influenza virus multiplication in cell cultures and that catechin has viable viricidal actions against a wide range of viruses, including those of the Flaviviridae, Orthomyxoviridae and Retroviridae families. Furthermore, EGCG acts against the human immunodeficiency virus (HIV) by inhibiting the enzymatic activities of the herpes simplex virus 1 and 2 (HSV-1 and HSV-2), hepatitis C virus (HCV) and HIV-1 reverse transcriptase (Liu et al., 2021). Based on the principle of reverse expression, catechin has the potential to prevent SARS-COV-2 from entering the CNS.

Therefore, understanding the expression patterns of ACE2 in the nervous system is crucial in determining the neural system’s vulnerability to SARS-CoV-2 infection. This study examines the expression of ACE2 and associated genes in brain tissues, aiming to elucidate the susceptibility of the CNS to SARS-CoV-2 infection.

## Materials and methods

### Datasets

A single-cell RNA-seq expression profile of the mouse brain vascular system was obtained from the Gene Expression Omnibus (GEO) repository (GSE60361). The gene expression levels in each cell were analysed. Genes with an expression level of less than 0.1% of the total number of cells in the study were excluded. Eventually, 3005 cell samples from the dataset were selected for analysis, and the samples satisfied the quality control standards.

### Clustering and dimensionality reduction

The Seurat package (version: 3.2.2) in the R software (version: 4.0.2) was utilized to conduct principal component analysis (PCA) using the PCEIbowPlot and JackStraw functions to identify key principal components (PCs). To determine gene heterogeneity in each cell group, the FindAllMarkers utility in Seurat was utilized. Following this, cell clustering and visual analysis of UMAP were performed utilizing the RunUMAP platform. The singleR package was subsequently utilized to annotate the marker genes, and CellMarker was thereafter employed to correct them based on their features.

### Pathway and process enrichment analysis

Pathway and process enrichment analysis was performed using Metascape (https://metascape.org/gp/index.html). Pathways and processes enrichment studies were performed using the following ontology resources for the ACE2-related gene list: PANTHER Pathway, WikiPathways, Transcription Factor Targets, PaGenBase, DisGeNET, TRRUST, CORUM, Canonical Pathways, Reactome Gene Sets, GO Biological Processes, KEGG Pathway and COVID. Moreover, all genes in the genome served as the enrichment background. To collect and classify terms based on their affiliation commonalities, a *p*-value less than 0.01, the least count of three and an enrichment factor of more than 1.5 (the enrichment factor denotes the ratio of the recorded counts and anticipated counts) were used. Furthermore, *p*-values were determined utilizing the accumulative hypergeometric distribution, whereas q-values were derived utilizing the Benjamini–Hochberg technique, which involves multiple tests. Kappa score was used for the hierarchical clustering of the enriched terms, wherein sub-trees with a similarity degree of more than 0.3 were deemed to be a cluster. The most significant statistical term inside a cluster was selected to serve as the cluster’s representative term (Hochberg and Benjamini, 1990).

### Protein-protein interaction enrichment analysis

To obtain the gene list of ACE2-related proteins, PPI enrichment analysis was performed utilizing different databases, includingInWeb_IM9, OmniPath8, BioGrid7, and STRING6 (Li et al., 2017; Oughtred et al., 2019; Szklarczyk et al., 2019). Furthermore, only the physical interaction function was used in STRING (with a physical score greater than 0.132) and BioGrid databases. The resulting network comprised the selection of proteins that have established physical interplays with a minimum of one other component on the list. When the networks consisted of approximately 3–500 proteins, the Molecular Complex Detection (MCODE) method was employed to determine the components of the network that significantly correlated with each other (Bader and Hogue, 2003).

### Small molecules identification

To predict relatively small active molecules that might attenuate the existing biological state of ACE2-related endothelial cells, an evaluation of the ACE2-related endothelial cells was performed *via* the comparison of the differentially expressed genes (DEGs) between clusters 4 and 13 against those found in the Connectivity Map database (CMap, http://www.broadinstitute.org/cmap/). Initially, the DEGs were classified into two groups, namely downmodulated and upmodulated groups. Subsequently, for gene set enrichment analysis (GSEA), different expression significant probesets were selected from each group and evaluated, resulting in enrichment scores that ranged from −1 to +1. Furthermore, small molecules with positive connectivity values close to +1 were found to drive gene expression in cluster 13, while those with negative connectivity values close to −1 showed increased similarities between genes and small molecules, which might attenuate cluster 4’s status.

### Human protein atlas database analysis

HPA (https://www.proteinatlas.org/) is a database containing details on cell and tissue distributions among the 24,000 proteins found in the human body. It employs specialised antibodies and immunohistochemical technologies to examine the dispersion and expression of every protein in 48 different types of normal human tissues, 12 different types of blood cells, 47 different types of cell lines and 20 different types of tumour tissues. These tissues are collected from 144 distinct normal and 216 distinct tumour tissues, thus guaranteeing that the immunohistochemical findings are representative of the population. Thus, using this database, both the prognostic value and protein expression levels of the most possible hub genes in brain tissues were validated.

### Immunofluorescence

Juvenile Sprague–Dawley (SD) rats aged 4 weeks and old SD rats aged 12 months were selected and subsequently treated with intracardiac perfusion of 0.1 mmol phosphate-buffered saline (PBS) and 4% paraformaldehyde. Eight-micrometre coronal cryosections were incubated and blocked with 5% bovine serum albumin (BSA) for 2 h. Frozen sections were then incubated overnight at 4°C with primary antibodies. The primary antibodies used were anti-CD31 antibody (1:2000, Abcam, ab9498) and anti-ACE2 antibody (1:1000, Proteintech, 21115-1-AP). After overnight incubation, frozen sections were incubated with fluorescent secondary antibody (1:2000, Abcam) at room temperature for 2 h. Then, the sections were washed thrice with PBS and covered with fluorescent fixation medium containing 4′,6-diamidino-2-phenylindole (DAPI) (1:1000, Solarbio). Image acquisition was performed using an Olympus fluorescence microscope with an eyepiece magnification of ×10 and an objective lens magnification of ×20. The exposure time of each section was 20 ms. Particle fluorescence intensity of ACE2 was calculated using ImageJ software (National Institutes of Health, United States) after filming, and each group included six animals.

### Western blot

Brain tissues from juvenile and aged SD rats were lysed in RIPA buffer (Solarbio, Beijing, China), following this protease and phosphatase inhibitors were added and then the sample was denatured at 100°C for 15 min. The protein samples were then separated using 10% sodium dodecyl-sulfate polyacrylamide gel electrophoresis and transferred to polyvinylidene fluoride (PVDF) membranes. Next, PVDF membranes were blocked with 5% skim milk powder solution for 1 h, incubated with primary antibodies, including anti-ACE2 antibody (1:1000, Proteintech, 21115-1-AP) and anti-Tubulin antibody (1:10000, Abmart, M20005) overnight, followed by secondary antibodies (1:5000) for 2 h at room temperature. The bands were visualised using an ECL kit chemiluminescence reagent (Billerica Millipore, MA, United States). Protein band signals were detected using the Chemidoc detection system (Bio-Rad, Hercules, CA, United States) and quantified by the ImageJ software (National Institutes of Health, United States).

## Results

### Utilization of scRNA-seq data to analyze and identify 15 cell clusters in brain tissues

**FIGURE 1:**
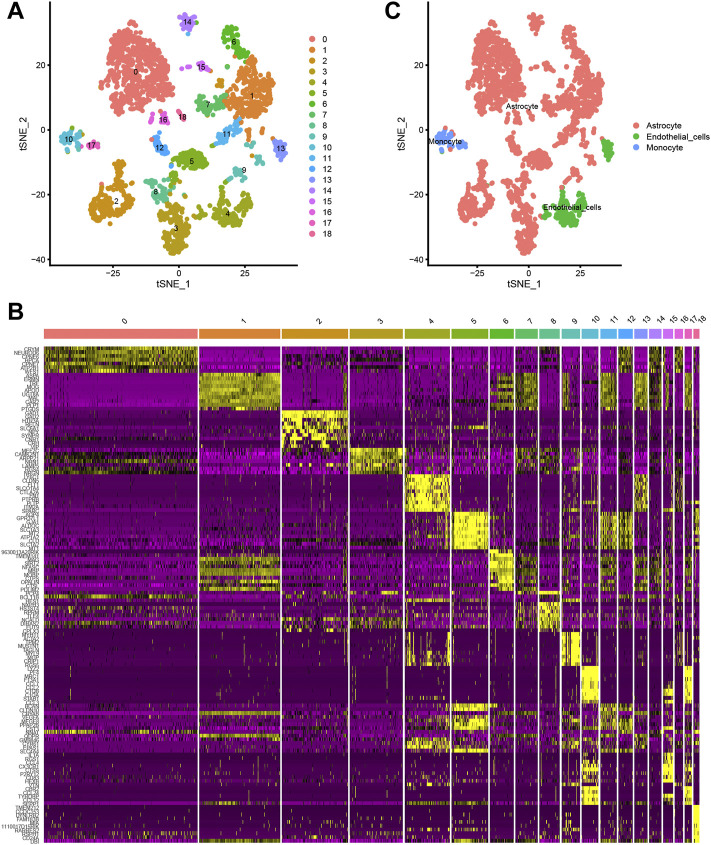
**(A, B)** The t-distributed stochastic neighbour embedding (t-SNE) technique classifies 18 cell clusters using the relevant PCs identified via principal component analysis. **(C)** A total of 18 clusters were identified using differential analysis. The top 10 marker genes in each cell cluster are shown in the heatmap.

A total of 3,005 cells from 67 mice were acquired in this study. t-distributed stochastic neighbour embedding (t-SNE) technique was then used to divide the cells into 18 distinct clusters (Figure 1A). Differential expression analysis facilitated the identification of marker genes of the 18 different cell clusters (|logFC| >1 and adjusted *p*-value < 0.05) (Figures 1B,C). Annotations of the types of cells in the 18 cell clusters were performed utilizing singleR and the CellMarker database (Figure 1D).

### Identification of two separate types of endothelial cells with unique biological functions and differentiation states

**FIGURE 2:**
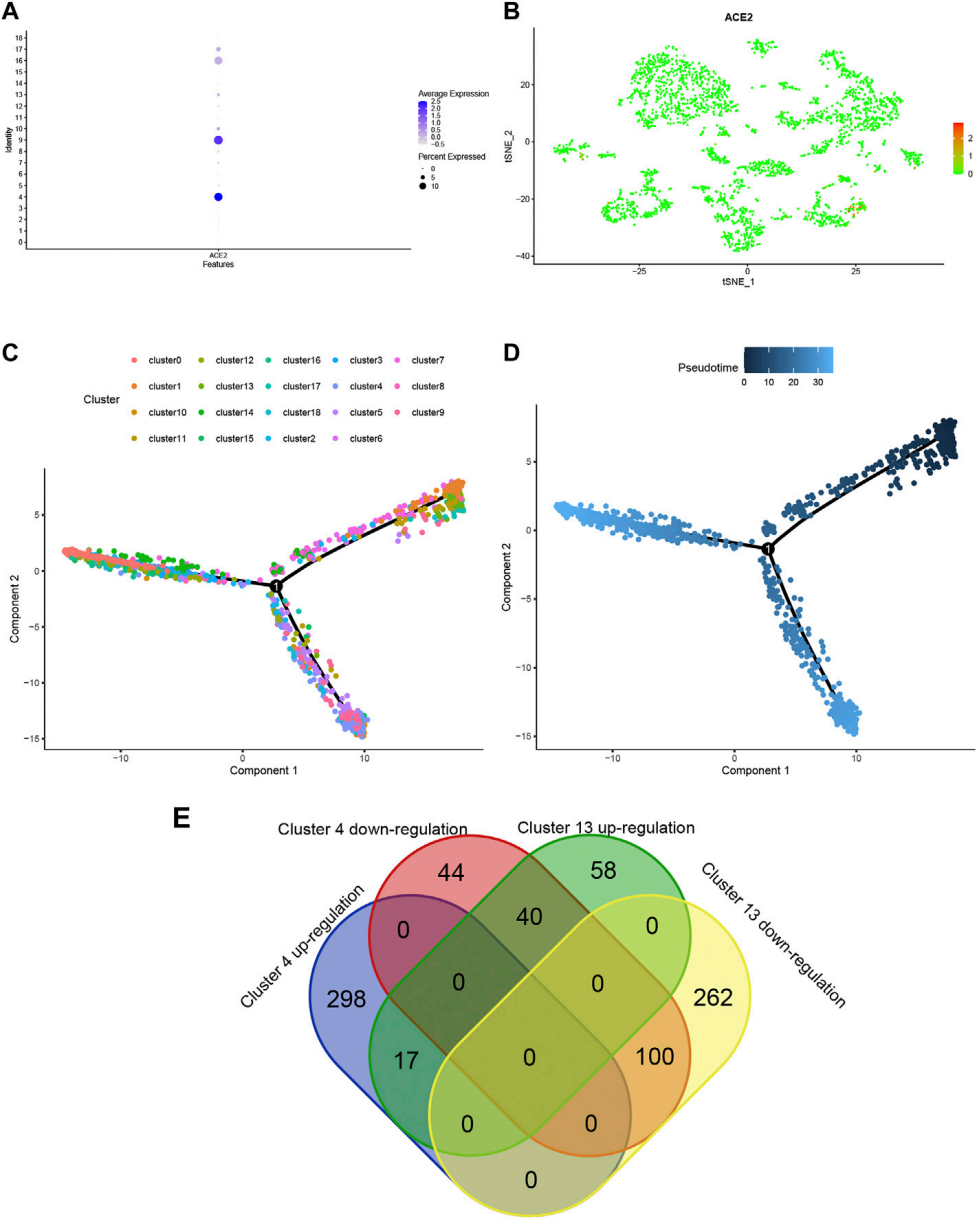
**(A)** Bubble plot of ACE2 expression in different cell clusters. **(B)** Dot plot shows ACE2 expression in each cell cluster. **(C,D)** The trajectory plot in pseudotime of each cell cluster using Monocle analysis. Different colours represent different cell states. **(E)** Venn plot of Cluster 4 and Cluster 13 in up- and down-regulated states.

Using ACE2 as the marker, individual brain cells were successfully identified. Cluster 4 (endothelial cells) showed the highest average expression of ACE2 (Figure 2A). The endothelial cells were classified into two groups, namely cluster 4 and cluster 13 (Figure 2B). Furthermore, a considerable differentiation propensity was observed between cluster 13 with low-ACE2-expression in the former branch and cluster 4 within the latter branch on performing pseudotime trajectory analysis. This suggests that ageing endothelial cells are more susceptible to the SARS-CoV-2 virus (Figures 2C,D).

### DEGs of cluster 4 and cluster 13

To explore the expression model of cluster 4 and cluster 13, a Venn qplot was drawn to show the up-regulated genes and downregulated genes in each cluster (Figure 2G). Genes with opposite expressions were considered to be DEGs in these two classifications.

### Enrichment analyses

**FIGURE 3:**
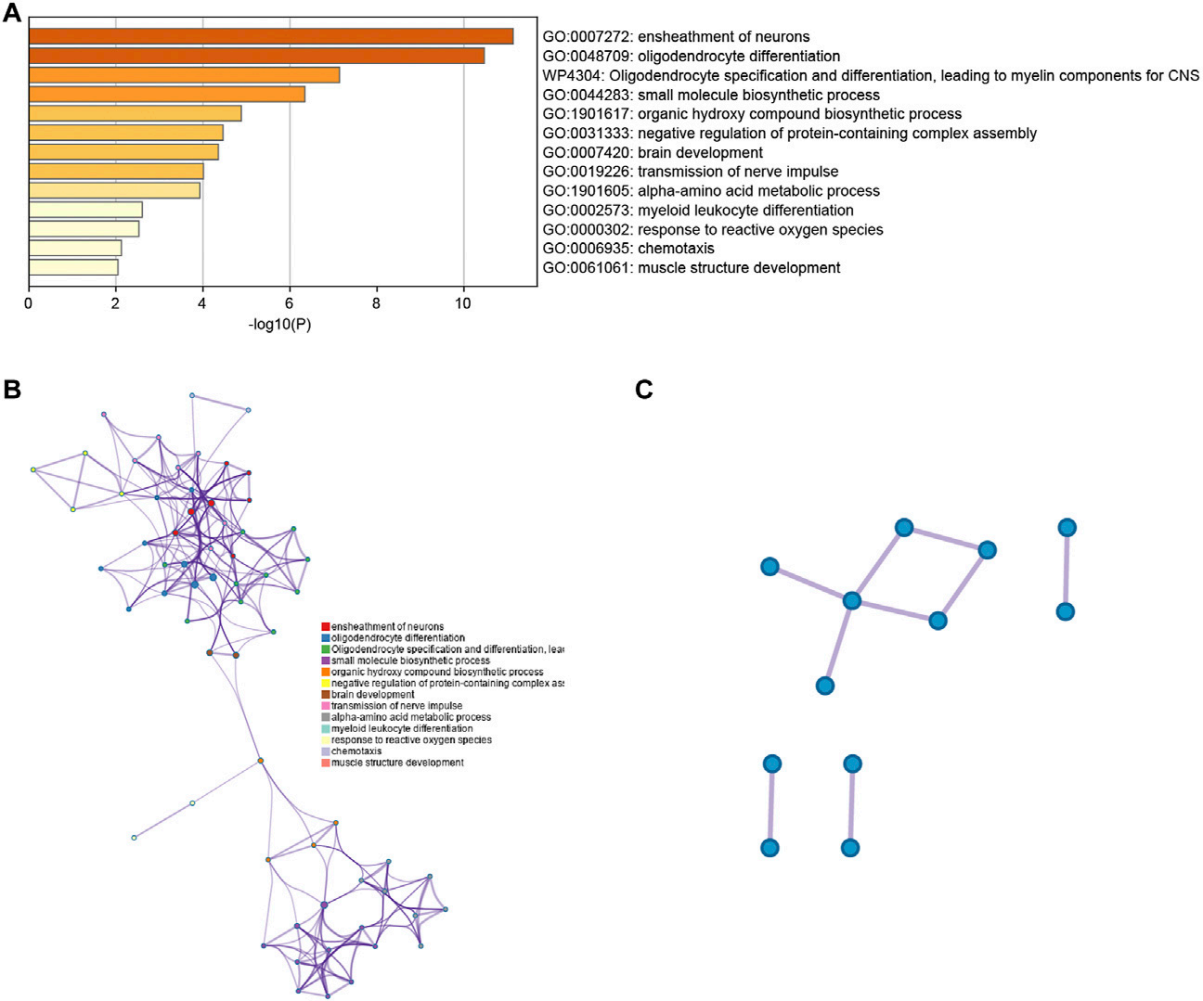
**(A)** Bar graph representing the enriched terms in the gene list, which are shaded according to their *p*-values. **(B)** A network of enriched terms, shaded according to cluster-ID, with nodes sharing a common cluster-ID often situated adjacent to each other. **(C)** The gene list reveals a network of protein-protein interactions and MCODE components.

For a thorough understanding of the biological mechanisms of DEGs between clusters 4 and 13, Metascape was used to conduct GO and KEGG pathway enrichment analyses. The DEGs were found to be mostly enriched in the ensheathment of neurons, oligodendrocyte differentiation and oligodendrocyte specification, leading to myelin components for CNS, small molecule biosynthetic process, and organic hydroxy compound biosynthetic process (Figures 3A,B).

### Establishment of the PPI network, module analysis and localization

**FIGURE 4:**
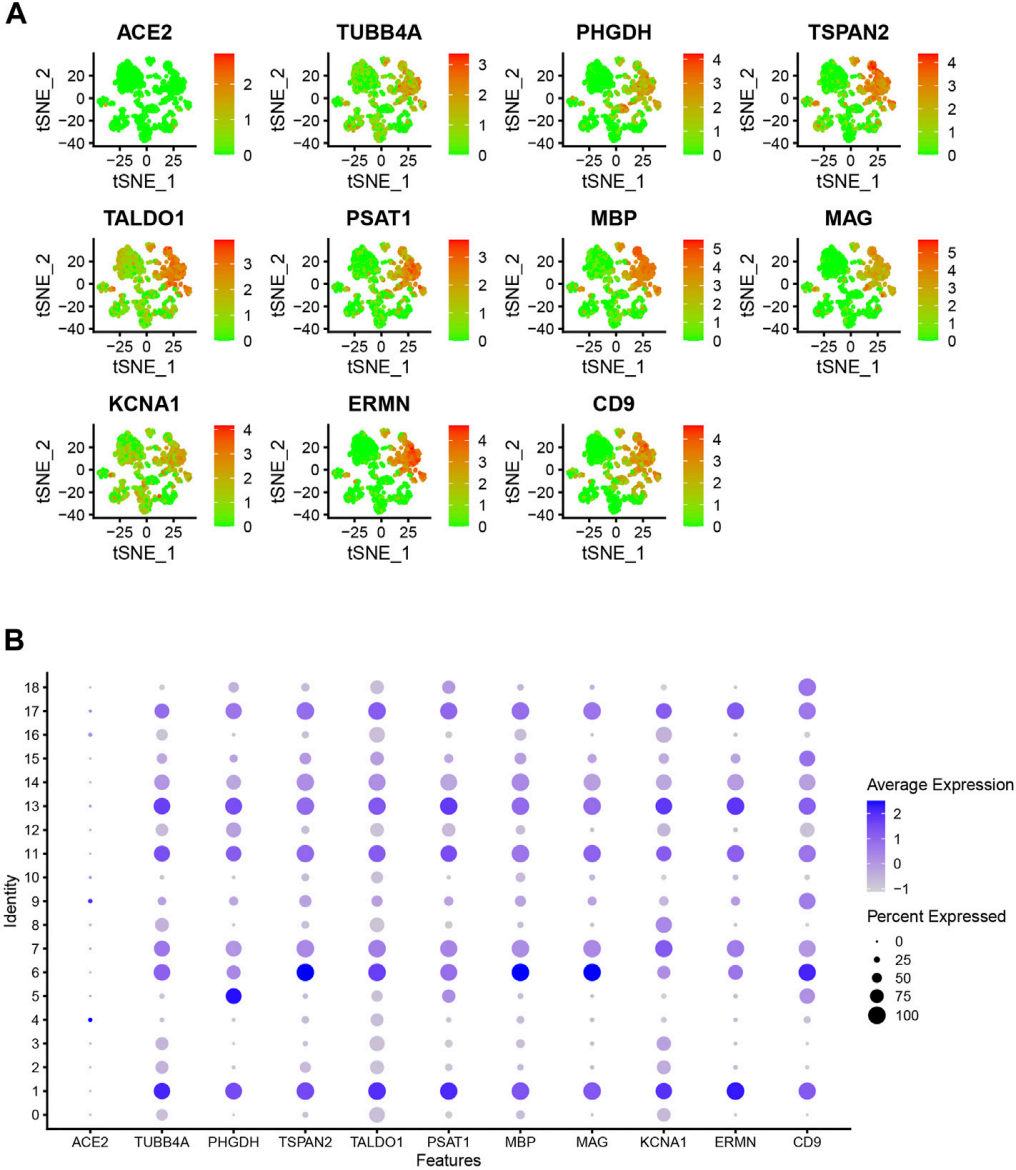
**(A)** Dot plot shows hub gene expressions in each cell cluster. **(B)** Bubble plot shows hub gene expressions in each cell cluster.

The PPI network for the DEGs and MCODE showed that TUBB4A, TSPAN2, TALDO1, SCD5, PSAT1, PHGDH, MSMO1, MBP, MAG, KCNA1, ERMN and CD9 play a key role in SARS-CoV-2 infection (Figure 3C). Moreover, the hub genes are highly expressed in some astrocytes. The interaction of endothelial cells and astrocytes can cause SARS-COV-2 to enter the CNS. The expression of these hub genes among cell subsets is shown in Figures 4A,B.

### Identification of related active small molecules

**FIGURE 5:**
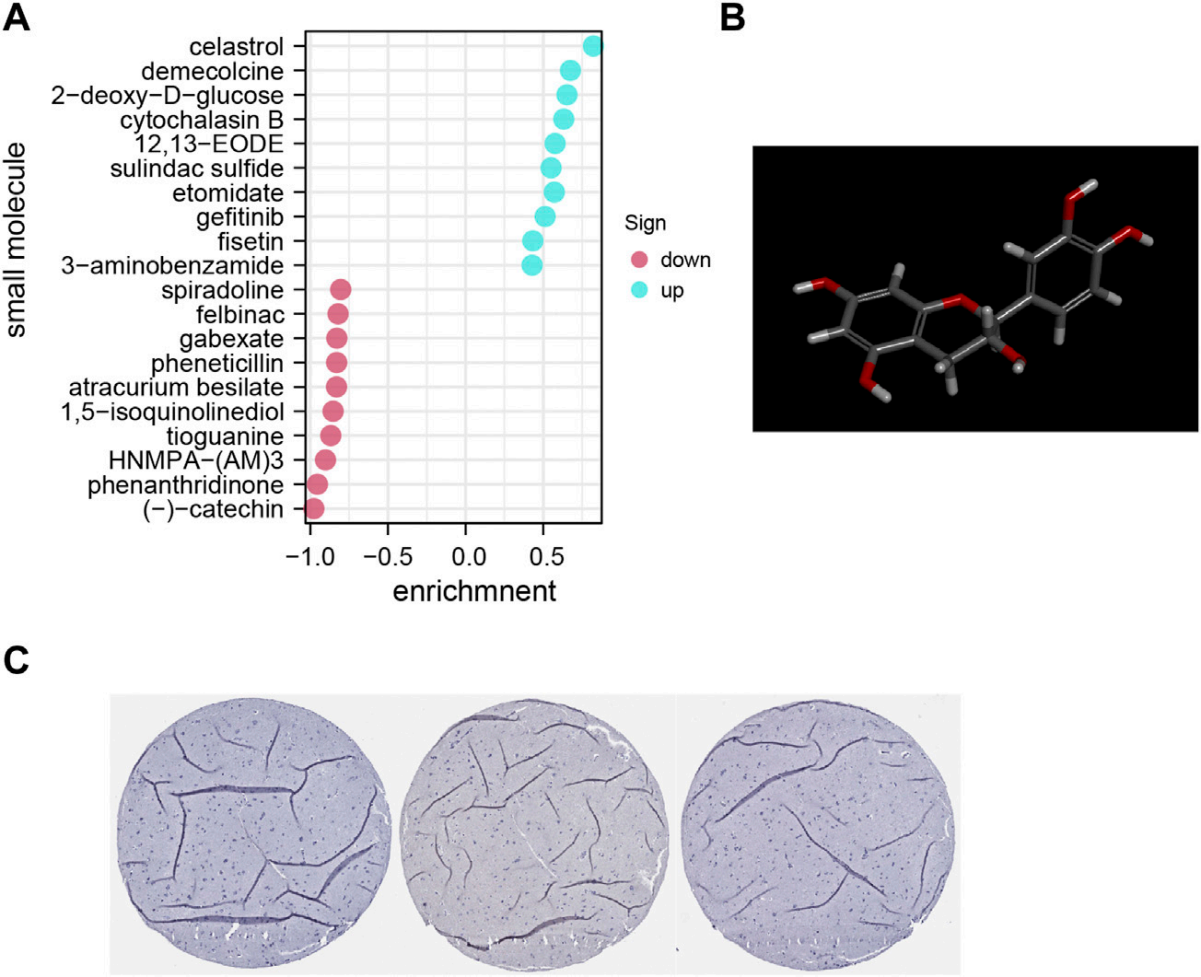
**(A)** Pop plot showing the top 20 small compounds capable of reversing gene expression. **(B)** Structure of top molecule. **(C)** Representative immunohistochemistry staining results reveal the protein level expression of ACE2 in brain tissues.

The DEG data that had been classified into the upmodulated and downmodulated groups were entered into the CMap database, where it was subjected to further integration with small molecule treatments to evaluate and identify potential therapeutic medicines for ACE2-related endothelial cells. Figure 5A illustrate the top 20 relevant small molecules and their enrichment scores, respectively. A significant negative score was found to be associated with the small molecules of phenanthridinone (enrichment score = −0.954) and (–)catechin (enrichment score = −0.977), suggesting that these molecules characterize cluster 13. These prospective small molecule medications have the capacity of attenuating gene expression, thereby identifying potential novel pathways and molecular processes for innovative targeted treatments focusing on the CNS. However, further research is required to determine the specific significance of these potential small compounds.

### ACE2 protein expression in the brain

**FIGURE 6:**
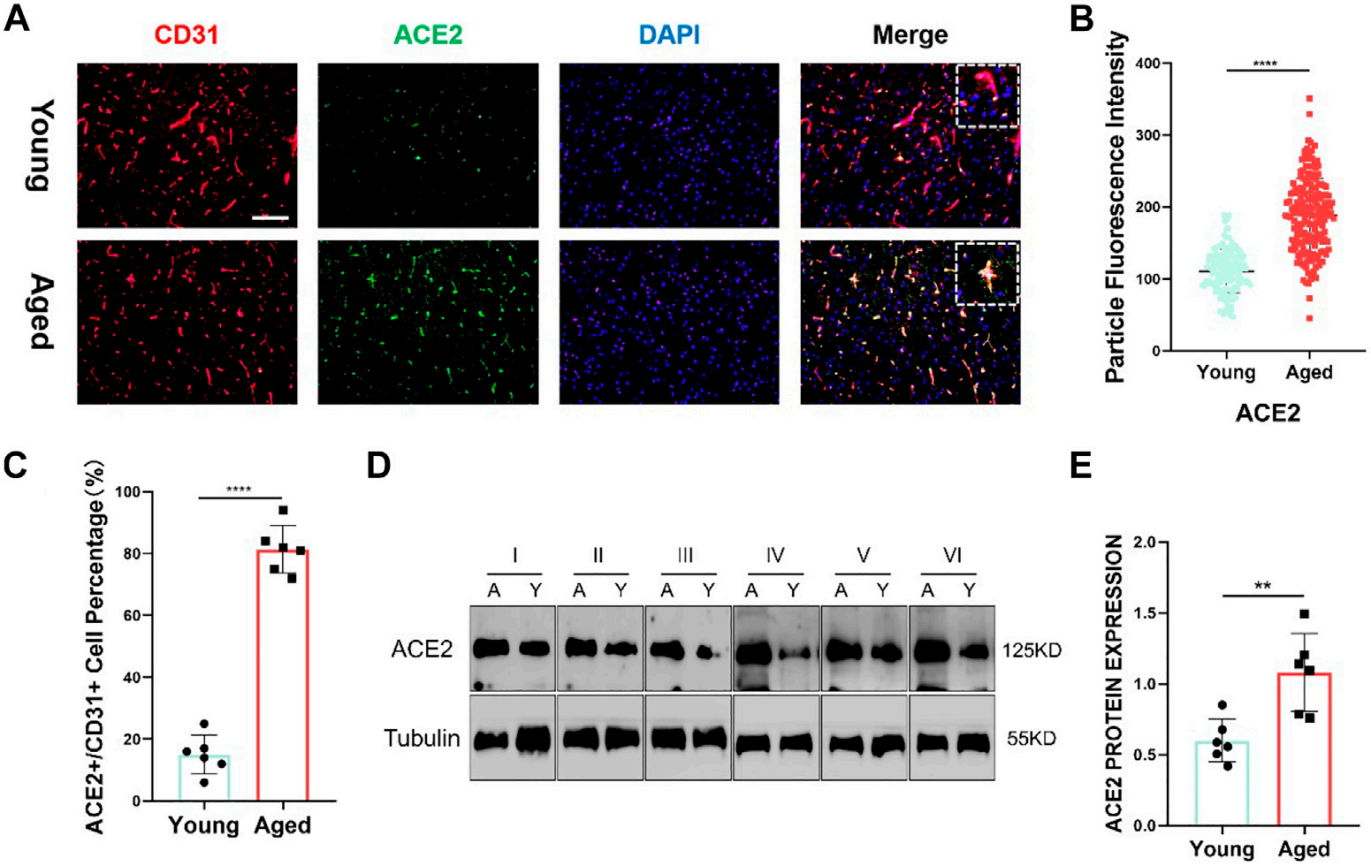
**(A)** Representative images of immunofluorescence staining for CD31 (red), ACE2 (green) and DAPI (blue) in the juvenile and aged Sprague–Dawley (SD) rats. Scale bar = 100μm; *n* = 6. **(B,C)** ACE2 shows a significant difference between the two groups. Data are presented as mean ± standard deviation (SD); **** *p*-value < 0.0001; *n* = 151 and 183 **(D)** Representative western blot images of ACE2 and tubulin from the two groups. *n* = 6. **(E)** Tubulin was used as a protein loading control; mean ± SD of eight independent experiments. **p-value < 0.01.

Furthermore, utilizing clinical samples from the HPA repository, the ACE2 protein expression level was determined. Immunohistochemical results showed that the gene was mainly expressed in cerebral vessels (Figure 5B). Representative images of immunofluorescence staining for CD31 (red), ACE2 (green) and DAPI (blue) in the young and aged SD rats are shown in Figure 6A. Additionally, immunofluorescence showed that ACE2 was mainly expressed in endothelial cells and was significantly highly expressed in the brain endothelium of aged rats compared to that of young rats (*p* < 0.0001; Figures 6B,C). Furthermore, the western blot results showed that ACE2 expression in the brain in aged rats was significantly higher than that in young rats (Figures 6C,D).

## Discussion

Respiratory viruses are capable of infecting the upper respiratory system in humans, resulting in mild illnesses in most cases (Gunathilake et al., 2021). However, in susceptible groups, such as neonates, infants, older adults and immunocompromised individuals, these pathogens may also impact the lower respiratory tract, resulting in more serious infections such as pneumonia (Desforges et al., 2019). Furthermore, due to the virus’s ability to adapt quickly and transcend the species barrier, most of these infections, including SARS-CoV and influenza A, have sometimes caused epidemics or pandemics. They have also been correlated with more significant clinical illnesses and even death (Berth et al., 2009). Additionally, various studies over decades have reported that certain respiratory viruses have neural-invasive abilities, indicating that they may migrate from the respiratory system into the CNS (Dahm et al., 2016b). Viruses that infect human CNS cells can subsequently induce various forms of encephalopathy, such as encephalitis and long-term neurologic illnesses. Although various therapeutic compounds are currently being investigated, there remains a scarcity of effective and reliable therapeutic regimens to treat SARS-CoV-2. Moreover, studies regarding SARS-CoV-2 in the CNS remain scarce.

Generally, an infection stimulates the endothelial cells to release chemokines, which improves vascular permeability and allows viruses to get through the first layer of the BBB (Mladinich et al., 2021). Furthermore, viruses commonly employ proteins produced by the endothelium and enter these cells. While SARS-Cov-2 infections are commonly limited to the airways, it has been reported to cross the epithelial barrier and infiltrate the CNS. This is consistent with the mechanism of other respiratory viral pathogens, such as influenza virus, Nipah virus and respiratory syncytial virus (RSV). In this study, brain endothelial cells showed significant expression levels of the enzyme ACE2. The SARS-CoV-2 virus enters the host cell via the SARS-CoV receptor ACE2. Hence, it was speculated that SARS-CoV-2 employs the ACE2 receptor for intracellular penetration into the CNS by infecting endothelial cells. The time analysis of cells showed that endothelial cells in the advanced stage had higher expressions of ACE2. This suggests that elderly patients are more likely to be infected by SARS-CoV-2 via the endothelial cells of the CNS.

Several patients with SARS-CoV-2 (i.e., who had a positive RT-PCR test) also experienced the loss of smell, despite not experiencing nasal obstruction dysgeusia, albeit exhibiting swelling in the olfactory cleft, which was validated using magnetic resonance imaging. The olfactory cleft is responsible for the flow of odours to the olfactory epithelium and then to the olfactory bulb. The olfactory epithelium (commonly referred to as the olfactory mucosa) consists of olfactory receptor neurons, basal cells and epithelial cells, all of which function together to create ‘smell’. When TNF-alpha (TNF-α) and interleukin-1 beta (IL1β) are released, the above cells react to create a “smell”. Notably, SARS-CoV-2 infection has been demonstrated to contribute to a higher production of TNF-α and IL1β. Consequently, the pathogenesis of SARS-CoV-2 could impact the lower respiratory tract while simultaneously impacting surrounding cells (such as those found in the respiratory tract), resulting in affecting the CNS.

Increasing evidence has identified the SARS-Cov-2 virus as the source of EGCG’s antiviral effects (Upadhyay et al., 2020) Furthermore, it has been shown that EGCG attenuated the enzymatic activities of the coronavirus 3CL protease, preventing the virus from replicating. Moreover, EGCG has the ability to control particular targets such as the RdRp and viral S protein. It has also been shown to be effective in preventing the reproduction of SARS-CoV-2 in cell incubation experiments. Molecular docking studies also show that EGCG inhibits SARS-CoV-2 entry into the target cell by interfering with the RBD in the viral membrane that binds to ACE2. This study suggests that EGCG could prevent SARS-CoV-2 from entering the CNS through endothelial cells by inhibiting its expression. In order to verify the utility of EGCG in anti-SARS-CoV-2 treatments, more pre-clinical investigations, clinical trials and epidemiological analyses are necessary.

The findings in the present research are restricted as only mouse tissue samples were used. Nonetheless, this study provides proof that SARS-CoV-2 could infiltrate the CNS through a large number of susceptible cells. Moreover, endothelial cells in elderly patients have a greater susceptibility to infection by SARS-CoV-2. Furthermore, the influence of SARS-COV-2 on the CNS requires more attention.

## Data Availability

The datasets presented in this study can be found in online repositories. The names of the repository/repositories and accession number(s) can be found in the article/Supplementary Material.

## References

[B1] Argyris E. G. Acheampong E. Wang F. Huang J. Chen K. Mukhtar M. (2007). The interferon-induced expression of APOBEC3G in human blood-brain barrier exerts a potent intrinsic immunity to block HIV-1 entry to central nervous system. Virology 367 (2), 440–451. 10.1016/j.virol.2007.06.010 17631933 PMC2737467

[B2] Atluri V. S. Hidalgo M. Samikkannu T. Kurapati K. R. V. Jayant R. D. Sagar V. (2015). Effect of human immunodeficiency virus on blood-brain barrier integrity and function: an update. Front. Cell. Neurosci. 9, 212. 10.3389/fncel.2015.00212 26113810 PMC4461820

[B3] Bader G. D. Hogue C. W. (2003). An automated method for finding molecular complexes in large protein interaction networks. BMC Bioinforma. 4, 2. 10.1186/1471-2105-4-2 PMC149346 12525261

[B4] Berth S. H. Leopold P. L. Morfini G. N. (2009). Virus-induced neuronal dysfunction and degeneration. Front. Biosci. 14, 5239–5259. 10.2741/3595 19482613

[B5] Bhattacharyya U. Thelma B. K. (2020). Age-related gene expression alterations by SARS-CoV-2 infection contribute to poor prognosis in elderly. J. Genet. 99 (1), 80. 10.1007/s12041-020-01233-7 33168795 PMC7584866

[B6] Boras B. Jones R. M. Anson B. J. Arenson D. Aschenbrenner L. Bakowski M. A. (2021). Preclinical characterization of an intravenous coronavirus 3CL protease inhibitor for the potential treatment of COVID19. Nat. Commun. 12 (1), 6055. 10.1038/s41467-021-26239-2 34663813 PMC8523698

[B7] Dahm T. Rudolph H. Schwerk C. Schroten H. Tenenbaum T. (2016). Neuroinvasion and inflammation in viral central nervous system infections. Mediat. Inflamm. 2016, 8562805. 10.1155/2016/8562805 PMC4897715 27313404

[B8] Dahm T. Rudolph H. Schwerk C. Schroten H. Tenenbaum T. (2016). Neuroinvasion and inflammation in viral central nervous system infections. Mediat. Inflamm. 2016, 8562805. 10.1155/2016/8562805 PMC4897715 27313404

[B9] Desforges M. Le Coupanec A. Dubeau P. Bourgouin A. Lajoie L. Dube M. (2019). Human coronaviruses and other respiratory viruses: Underestimated opportunistic pathogens of the central nervous system? Viruses 12 (1), E14. 10.3390/v12010014 31861926 PMC7020001

[B10] Gunathilake T. Ching Y. C. Uyama H. Chuah C. H. (2021). Nanotherapeutics for treating coronavirus diseases. J. Drug Deliv. Sci. Technol. 64, 102634. 10.1016/j.jddst.2021.102634 34127930 PMC8190278

[B11] Hochberg Y. Benjamini Y. (1990). More powerful procedures for multiple significance testing. Stat. Med. 9 (7), 811–818. 10.1002/sim.4780090710 2218183

[B12] Ladner J. T. Larsen B. B. Bowers J. R. Hepp C. M. Bolyen E. Folkerts M. (2020). An early pandemic analysis of SARS-CoV-2 population structure and dynamics in Arizona. mBio 11 (5), e02107-20. 10.1128/mBio.02107-20 32887735 PMC7474171

[B13] Lambertini M. Patrizi A. Peris K. Marasco G. Toselli M. Marcelli E. (2021). The impact of the COVID-19 pandemic on dermatologic practice: an Italian survey. Eur. J. Dermatol. 31 (1), 55–59. 10.1684/ejd.2021.3970 33648913 PMC8120755

[B14] Li T. Wernersson R. Hansen R. B. Horn H. Mercer J. Slodkowicz G. (2017). A scored human protein-protein interaction network to catalyze genomic interpretation. Nat. Methods 14 (1), 61–64. 10.1038/nmeth.4083 27892958 PMC5839635

[B15] Liu S. Y. Wang W. Ke J. P. Zhang P. Chu G. X. Bao G. H. (2021). Discovery of Camellia sinensis catechins as SARS-CoV-2 3CL protease inhibitors through molecular docking, intra and extra cellular assays. Phytomedicine. 96, 153853. 10.1016/j.phymed.2021.153853 34799184 PMC8575542

[B16] Mavian C. Marini S. Prosperi M. Salemi M. (2020). A snapshot of SARS-CoV-2 genome availability up to april 2020 and its implications: Data analysis. JMIR public health surveillance 6 (2), e19170. 10.2196/19170 32412415 PMC7265655

[B17] Mladinich M. C. Conde J. N. Schutt W. R. Sohn S. Y. Mackow E. R. (2021). Blockade of autocrine CCL5 responses inhibits zika virus persistence and spread in human brain microvascular endothelial cells. mBio 12 (4), e0196221. 10.1128/mBio.01962-21 34399621 PMC8406327

[B18] Moriguchi T. Harii N. Goto J. (2020). A first case of meningitis/encephalitis associated with SARS-Coronavirus-2. Int. J. Infect. Dis. IJID 94, 55–58. 10.1016/j.ijid.2020.03.062 32251791 PMC7195378

[B19] Omolaoye T. S. Adeniji A. A. Cardona Maya W. D. du Plessis S. S. (2021). SARS-COV-2 (Covid-19) and male fertility: Where are we? Reprod. Toxicol. 99, 65–70. 10.1016/j.reprotox.2020.11.012 33249233 PMC7689309

[B20] Oughtred R. Stark C. Breitkreutz B. J. Rust J. Boucher L. Chang C. (2019). The BioGRID interaction database: 2019 update. Nucleic Acids Res. 47 (D1), D529-D541–d541. 10.1093/nar/gky1079 30476227 PMC6324058

[B21] Schwerk C. Tenenbaum T. Kim K. S. Schroten H. (2015). The choroid plexus-a multi-role player during infectious diseases of the CNS. Front. Cell. Neurosci. (80), 80. 10.3389/fncel.2015.00080 25814932 PMC4357259

[B22] Szklarczyk D. Gable A. L. Lyon D. Junge A. Wyder S. Huerta-Cepas J. (2019). STRING v11: Protein-protein association networks with increased coverage, supporting functional discovery in genome-wide experimental datasets. Nucleic Acids Res. 47 (D1), D607-D613–d613. 10.1093/nar/gky1131 30476243 PMC6323986

[B23] Upadhyay S. Tripathi P. K. Singh M. Raghavendhar S. Bhardwaj M. Patel A. K. (2020). Evaluation of medicinal herbs as a potential therapeutic option against SARS-CoV-2 targeting its main protease. Phytother. Res. 34 (12), 3411–3419. 10.1002/ptr.6802 32748969 PMC7436756

[B24] Wang Y. Q. Li Q. S. Zheng X. Q. Lu J. L. Liang Y. R. (2021). Antiviral effects of green tea EGCG and its potential application against COVID-19. Mol. (Basel, Switz. 26 (13), 3962. 10.3390/molecules26133962 PMC8271719 34209485

