# Supplementary figures and images for: Single-cell sequencing of brain tissues reveal the central nervous system’s susceptibility to SARS-CoV-2 and the drug
Source: Front Pharmacol. 2022 Sep 13;13:971017. doi: 10.3389/fphar.2022.971017 (PMC9513673; doi:10.3389/fphar.2022.971017)

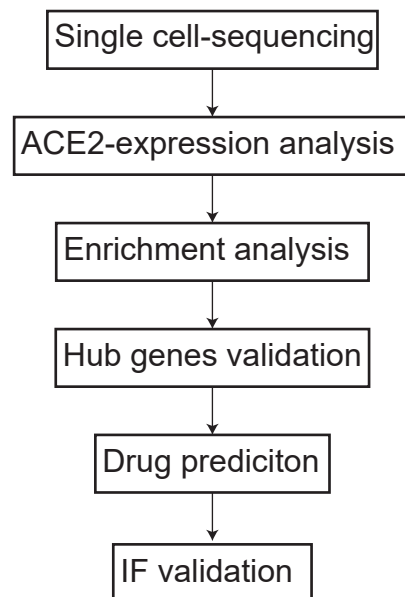

Supplement: Supplementary file 1 [file DataSheet1.PDF]
